# Familial correlation and aggregation of body mass index and blood pressure in Chinese Han population

**DOI:** 10.1186/1471-2458-13-686

**Published:** 2013-07-26

**Authors:** Yang Hu, Liu He, Yangfeng Wu, Guansheng Ma, Liming Li, Yonghua Hu

**Affiliations:** 1 Department of Epidemiology, Harvard University, Boston, USA; 2 Chinese Center for Disease Control and Prevention, Beijing, China; 3 Department of Epidemiology and Biostatistics, Peking University Health Science Center, Beijing, China; 4 National Institute for Nutrition and Food Safety, Chinese Center for Disease Control and Prevention, Beijing, China; 5 Department of Epidemiology and Biostatistics, Peking University Health Science Center, 38 Xueyuan Road, Beijing 100191, China

**Keywords:** Body mass index, Blood pressure, Family aggregation, Parent-offspring correlation, Sex-specific

## Abstract

**Background:**

It remains unclear whether the body mass index (BMI) and blood pressure (BP) profile are clustered within families in Chinese Han population. The aim of this study is to explore familial aggregation and parent-offspring correlations of BMI and blood pressure in Chinese Han population.

**Methods:**

6,369 Han nucleus families, consisting of parents and at least one biological adult child who were living together, were enrolled from the nation-wide cross-sectional study (China National Nutrition and Health Survey) which was conducted in 2002, with a total number of 19,107 participants aged 18–64 years (6,369 sets of parents, 4,132 sons and 2,237 daughters). Family aggregation (Intra-class correlations, ICCs) and parent-offspring correlations in BMI, systolic BP (SBP) and diastolic BP (DBP) were estimated using linear mixed effect regression models.

**Results:**

BMI and BP levels in two generations and ICCs of BMI, SBP and DBP varied across the country. Familial aggregation of overweight/obesity was observed in rural area (ICC = 5.4%, *p*<0.05), and high BP (defined as SBP ≥ 120 mmHg or DBP ≥ 80 mmHg) was more common in low income families (ICC = 4.4%, *p*<0.05) compared to middle income (ICC = 1.9%) and high income families (ICC = 2.6%). Additionally, offspring with more parents being overweight/obese tend to have higher BMI. The similar trend was found for high BP. However, we did not observe that same-sex parent-offspring correlations of BMI and BP were stronger than the correlations for mother-son or father-daughter.

**Conclusions:**

Our study suggested that familial environments, alongside the impact of genetic factors, could be important non-communicable chronic diseases (NCD) risk factors. Family-based intervention taking both mother and father into account might have great potential in NCD prevention for younger generation.

## Background

Obesity and hypertension are leading risk factors for non-communicable chronic diseases (NCDs) [1-4]. The prevalence of obesity is increasing at an alarming rate in economically developed countries as well as developing countries including China [5-7]. As body mass index (BMI) and blood pressure (BP) are important modifiable indicators for chronic diseases, interventions against obesity and hypertension are recognized to be effective for NCD prevention.

There is an increasing interest in the parental effect on their children with respect to BMI and BP, attributing to the impact of both genetic and environmental factors. Besides the strong impacts of parental BMI and BP on their offspring [8,9], recent findings of sex-specific associations suggest that childhood obesity is related to obesity of the same-sex parent [10,11], which was considered more likely to have an environmental than a genetic basis because selective mother–daughter and father–son gene transmission is not a common Mendelian trait [10]. A recent UK cohort study found that familial influence on BMI among middle-aged women appeared to be stronger from mothers than fathers [12]. However, argument still exists on this hypothesis [11], and findings of BP from an Indian population demonstrated father-offspring correlation of BP was stronger than mother-offspring correlation [13]. Since few studies with large sample size of nucleus families have been conducted in Asian populations, we performed an analysis to investigate whether there is familial aggregation of BMI and BP in Chinese Han population.

Using a large two-generational sample of 6,369 Han nucleus families across all 31 provinces, autonomous regions, and municipalities in China, our study assessed BMI and BP distribution among parents and offspring, and explored their familial aggregation. Since income levels are associated with BMI and BP [14], and modernization level is a good proxy for social economics status, we conducted the analysis stratified by different income levels or different modernization levels to minimize residual confounding. The stratification analysis would also enable us to investigate the distribution of familial aggregation status in different social economic status population. We also studied their same-sex and cross-sex parent-offspring correlations.

## Methods

### Study participants

In this study, Han nucleus families, consisting of parents and at least one biological child who were living together, were enrolled from the China National Nutrition and Health Survey 2002. This national survey was conducted from August to December 2002 with a well-designed stratified multistage cluster sampling method, and covered all 31 provinces, autonomous regions, and municipalities directly under the central government throughout China (except Taiwan, Hong Kong and Macao) [15]. Participants who were under 18 years old or over 65 years were excluded from the analysis. Among 732 families with more than one adult child, only their first one biological child was enrolled in this study, assuming they might be subject to more influence from their parents. 6,369 Han nucleus families with a total number of 19,107 participants, including 6,369 fathers, 6,369 mothers, 4,132 adult sons and 2,237 adult daughters, were included in the current analysis. Ethics approval was obtained from the Ethics Committee of China Center for Disease Control. All participants had signed on the informed consent.

### Survey methods

All participants were interviewed at a certain survey site in their living area. In order to reduce regional variance caused by temperature, surveys were run firstly in the north of China at an earlier time interval of the study, and then in the south. Information that was analyzed in this study was obtained by questionnaire and physical examination. Age, education level, occupation, and family annual per-capita income level information were collected by trained investigators through a questionnaire-based interview.

Height, weight and BP, were measured by well-trained and certified observers in the physical examination. Height was measured without shoes by a fixed stadiometer, and weight was measured with indoor clothes by traditional scales. BMI was calculated as the ratio of weight to height squared (kg/m^2^), overweight was defined as BMI ≥ 25 kg/m^2^ and obesity as BMI ≥ 30 kg/m^2^. BP was measured twice at right brachial artery according to 1999 World Health Organization/International Society of Hypertension guidelines on hypertension [16]. In this study, mean systolic BP (SBP) and diastolic BP (DBP) were used for analysis, and participants were classified as normotension (SBP<120 mmHg and DBP<80 mmHg), prehypertension (SBP of 120–139 mmHg or DBP of 80–89 mmHg), stage 1 hypertension (SBP of 140–159 mmHg or DBP of 90–99 mmHg) and stage 2 hypertension (SBP ≥ 160 mmHg or DBP ≥ 100 mmHg), according to the Seventh Joint National Committee on the Prevention, Detection, Evaluation and Treatment of hypertension (JNC-7) [17].

### Statistical methods

Continuous measures were shown as mean ± standard deviation (SD), and categorical variables were shown as percentages. Paired-t test and McNemar test were performed in comparison between fathers and mothers, while two-sample t test and Chi-square test were used to compare characteristics between groups of sons and daughters. Moreover, all individuals were divided into three groups either by family annual per-capita income (Group A: ≤1999 yuan; group B: 2000 ~ 4999 yuan; group C: ≥5000 yuan) or regions (Group A: major cities; group B: small or medium-sized cities; group C: rural areas). One way ANOVA was used to compare BMI, SBP, DBP levels of each family member by groups, and to avoid type I error, Student-Newman-Keuls (SNK) method was applied during the process of multiple comparisons between each two groups. This part of data analyses was conducted using the Statistical Package for the Social Science version 15.0 (SPSS Inc., Chicago, IL, USA).

Family aggregation (Intra-class correlations, ICCs) and parent-offspring correlations in BMI, SBP and DBP were estimated using multilevel linear regression models. With respect to acquirement of ICCs, three-level linear models were fitted across all individuals with separate area-level and family-level random intercepts, adjusting for individual age, education level and occupations, and ICCs were estimated as proportions of family-level variance to total variance (including area-level, family-level and individual-level variance) for BMI, SBP and DBP. Father/mother-son and father/mother-daughter correlations for BMI, SBP and DBP were estimated in 4,132 sons and 2,237 daughters separately, using two-level linear models fitted with separate area-level random intercepts, in which father’s or mother’s BMI, SBP or DBP were included as variables and child’s age, parental education level and occupations were considered as important covariates. Parent-offspring correlations were expressed as coefficients of father/mother’s BMI, SBP or DBP and their 95% confident interval. Multilevel analyses were performed using the software packages MLwiN version 2.1 [18].

All tests for statistical significance were two-sided and the significance level was set as α = 0.05.

## Results

Among 19,107 participants, comprising of 6,369 sets of parents, 4,132 sons and 2,237 daughters, characteristics specified by family members are shown in Table 1. Compared to mothers, fathers had higher average age, DBP levels, and higher proportion of prehypertension/hypertension, but lower BMI levels and lower overweight/obesity rate (*p*<0.001). And sons had higher age, BMI, SBP, DBP, and higher proportion of overweight/obesity and prehypertension/hypertension than daughters (*p*<0.001).

**Table 1:** Characteristics of family members, specified by generation and gender

	**Fathers**	**Mothers**	*p *value*	**Sons**	**Daughters**	***p *value#**
Number of individuals						
Total	6369	6369		4132	2237	
18 ~ 25 years	0	0		2533	1717	
26 ~ 35 years	3	8		1474	469	
36 ~ 45 years	953	1498		119	48	
46 ~ 55 years	3578	3757		6	3	
56 ~ 65 years	1835	1106		0	0	
Age (mean ± SD)	52.0 ± 6.2	49.8 ± 5.8	0.001	24.8 ± 5.0	23.0 ± 4.7	0.001
Body mass index (mean ± SD)	23.4 ± 3.3	24.2 ± 3.6	0.001	22.4 ± 3.5	21.6 ± 3.2	0.001
Systolic blood pressure (mean ± SD)	126.8 ± 19.4	126.2 ± 21.4	0.141	115.7 ± 12.1	108.1 ± 11.6	0.001
Diastolic blood pressure (mean ± SD)	81.6 ± 11.7	79.8 ± 11.8	0.001	75.1 ± 9.4	70.5 ± 8.7	0.001
Highly educated (High school and above) (%)	22.2	13.6	0.001	38.0	50.4	0.001
Employed (%)	78.9	53.0	0.001	89.8	80.3	0.001
Body weight classification (%)			0.001			0.001
Normal weight	69.8	62.6		79.4	87.8	
Overweight	27.0	30.8		17.5	10.1	
Obesity	3.2	6.6		3.1	2.1	
Blood pressure classification (%)			0.001			0.001
Normotension	28.7	35.0		51.2	75.7	
Prehypertension	41.4	36.8		41.1	21.3	
Stage 1 hypertension	19.1	17.5		6.5	2.6	
Stage 2 hypertension	10.8	10.7		1.2	0.3	

*Compared between paired fathers and mothers; # compared between grouped sons and daughters.

Table 2 shows that mean BMI, SBP and DBP of the two generations varied by family annual per-capita income and regional modernization level. Fathers, mothers and sons living in families with high income (family annual per-capita income ≥5000 yuan) or in major cities had the highest BMI levels, while those living in families with low income (family annual per-capita income <1999 yuan) or in rural areas had the lowest BMI levels. In addition, SBP and DBP levels of fathers were higher among those living in medium (family annual per-capita income 2000 ~ 4999 yuan) and high-income families, or in cities, while sons’ DBP level was higher in major cities. However, mothers’ SBP and DBP levels did not differ much among different family groups, and the daughters got lowest BMI and SBP level in small/medium-sized cities, and lowest BMI, SBP and DBP levels in high income families.

**Table 2:** Levels (means ± standard deviations) of BMI, SBP and DBP of family members, stratified by family annual per-capita income levels and regions

		**Family annual per-capita income level**				**Regions**			
		**≤1999 yuan (Group A)**	**2000 - 4999 yuan (Group B)**	**≥5000 yuan (Group C)**		**Major cities (Group A)**	**Small or medium-sized cities (Group B)**	**Rural areas (Group C)**	
		**(Nf = 2618)**	**(Nf = 1892)**	**(Nf = 1859)**		**(Nf = 1518)**	**(Nf = 889)**	**(Nf = 3962)**	
BMI	Fathers	22.7 ± 3.1	23.6 ± 3.2	24.3 ± 3.3	^abc^	24.6 ± 3.4	23.9 ± 3.2	22.9 ± 3.3	^abc^
	Mothers	23.7 ± 3.7	24.3 ± 3.8	24.6 ± 3.4	^abc^	25.0 ± 3.6	24.6 ± 3.4	23.7 ± 3.6	^abc^
	Sons	22.0 ± 3.1	22.4 ± 3.6	23.0 ± 3.8	^abc^	23.2 ± 4.3	22.7 ± 3.5	22.0 ± 3.5	^abc^
	Daughters	21.8 ± 3.0	21.6 ± 3.2	21.2 ± 3.3	^bc^	21.7 ± 3.8	21.0 ± 2.7	21.6 ± 3.2	^ab^
SBP	Fathers	125.6 ± 19.1	127.2 ± 19.8	127.7 ± 19.3	^ac^	128.2 ± 19.7	127.2 ± 18.6	126.1 ± 19.4	^c^
	Mothers	126.6 ± 22.3	125.7 ± 21.0	126.2 ± 20.6		126.0 ± 20.6	125.4 ± 20.6	126.5 ± 22.0	
	Sons	115.5 ± 12.0	115.9 ± 12.6	115.6 ± 11.6		115.5 ± 12.3	115.0 ± 12.0	115.9 ± 12.0	
	Daughters	109.3 ± 12.3	108.2 ± 11.2	106.5 ± 11.6	^bc^	107.0 ± 11.6	106.2 ± 11.7	109.2 ± 11.6	^bc^
DBP	Fathers	80.8 ± 11.6	81.7 ± 11.7	82.7 ± 11.5	^abc^	83.0 ± 11.7	82.9 ± 11.3	80.8 ± 11.6	^bc^
	Mothers	80.0 ± 12.1	79.4 ± 11.8	79.8 ± 11.2		80.0 ± 11.3	79.6 ± 11.6	79.7 ± 12.0	
	Sons	75.0 ± 9.3	75.1 ± 9.9	75.3 ± 8.9		75.8 ± 9.2	75.5 ± 9.6	74.8 ± 9.3	^c^
	Daughters	71.1 ± 8.9	70.6 ± 8.8	69.7 ± 8.4	^c^	70.7 ± 8.4	69.6 ± 8.9	70.7 ± 8.8	

Nf represents number of families.

During the process of multiple comparisons by SNK method, ‘a’ refers to *p*< 0.05 when compared between group A and B; ‘b’ refers to *p*< 0.05 when compared between group B and C; ‘c’ refers to *p*< 0.05 when compared between group A and C.

Family aggregation of BMI and BP are presented in Table 3. After controlling for age and individual socioeconomic status (education level and occupation), statistically significant ICCs of BMI, SBP and DBP were shown in families with different income levels or families in different regions, except for BMI in small and medium-sized cities and SBP in medium income families. In addition, familial aggregation of overweight/obesity was observed in rural areas, while those of high BP were clustered in families with low annual income and in regions of all 3 regions.

**Table 3:** Adjusted ICCs of body mass and blood pressure related indicators, stratified by family annual per-capita income levels and regions#

		**BMI**	**SBP**	**DBP**	**BMI ≥ 25 kg/m**^**2**^	**SBP ≥ 120 mmHg or DBP ≥ 80 mmHg**
Family annual per-capita income level						
	≤1999 yuan (Nf = 2618)	6.5%*	8.3%*	11.7%*	3.8%	4.4%*
	2000 - 4999 yuan (Nf = 1892)	7.4%*	4.7%	9.0%*	1.5%	1.9%
	≥5000 yuan (Nf = 1859)	6.6%*	8.8%*	7.8%*	2.5%	2.6%
Regions						
	Major cities (Nf = 1518)	6.9%*	6.3%*	6.2%*	3.2%	4.1%*
	Small and medium-sized cities (Nf = 889)	3.8%	6.5%*	9.1%*	4.9%	6.0%*
	Rural areas (Nf = 3962)	6.8%*	7.1%*	8.2%*	5.4%*	3.4%*

# Age, education levels and occupations were adjusted.

*p< 0.05.

Families were also classified into four categories either by parents’ overweight/obesity status or by parents’ high BP status. BMI, SBP and DBP levels of sons and daughter in each classification of families with adjustment of children’s age, education level, occupation, family annual per-capita income levels and regions, were shown separately in Table 4. Adjusted BMI, SBP and SBP levels of sons and daughters with both their parents’ BMI ≥ 25 kg/m^2^ were higher than those from families having only one overweight/obese parent, and much higher than those with both parents who had normal weight. In respect to families with only one overweight/obese parent, children’s BMI, SBP and DBP levels did not differ much according to the overweight/obese parent’s gender. Regarding parental high BP status, both sons’ and daughters’ SBP and DBP levels showed increasing trends with number of high-blood-pressure parents increased; however, BMI levels did not vary much among these four family categories.

**Table 4:** Adjusted means ± standard deviations of child’s BMI, SBP and DBP according to different family classifications*

	**Son**				**Daughter**			
	**Nf**	**BMI**	**SBP**	**DBP**	**Nf**	**BMI**	**SBP**	**DBP**
Families classified by parental BMI								
Both parents’ BMI<25 kg/m^2^	1990	21.4 ± 0.1	114.4 ± 0.3	74.1 ± 0.2	987	20.8 ± 0.1	107.4 ± 0.4	69.8 ± 0.3
Only father’s BMI ≥ 25 kg/m^2^	638	22.9 ± 0.1	116.3 ± 0.5	75.2 ± 0.4	366	22.0 ± 0.2	108.4 ± 0.6	70.8 ± 0.4
Only mother’s BMI ≥ 25 kg/m^2^	925	22.9 ± 0.1	116.0 ± 0.4	75.6 ± 0.3	533	21.7 ± 0.1	108.3 ± 0.5	70.9 ± 0.4
Both parents’ BMI ≥ 25 kg/m^2^	570	24.5 ± 0.1	119.0 ± 0.5	77.7 ± 0.4	347	23.0 ± 0.2	109.3 ± 0.6	71.8 ± 0.5
Families classified by parental blood pressure								
Both parents’ BP<120/80 mmHg	473	21.8 ± 0.2	111.0 ± 0.6	71.9 ± 0.4	292	20.8 ± 0.2	104.2 ± 0.6	67.2 ± 0.5
Only father’s BP ≥ 120/80 mmHg	929	22.3 ± 0.1	114.7 ± 0.4	74.3 ± 0.3	530	21.4 ± 0.1	107.0 ± 0.5	69.5 ± 0.4
Only mother’s BP ≥ 120/80 mmHg	701	22.2 ± 0.1	113.7 ± 0.4	73.6 ± 0.3	359	21.3 ± 0.2	106.6 ± 0.6	69.4 ± 0.4
Both parents’ BP ≥ 120/80 mmHg	2020	22.7 ± 0.1	117.9 ± 0.3	76.8 ± 0.2	1052	21.9 ± 0.1	110.2 ± 0.4	72.3 ± 0.3

*Means and standard deviations had been adjusted for children’s age, education level, occupation, family annual per-capita income levels and regions.

Nf represents number of families; BP refers to blood pressure.

**Figure 1:**
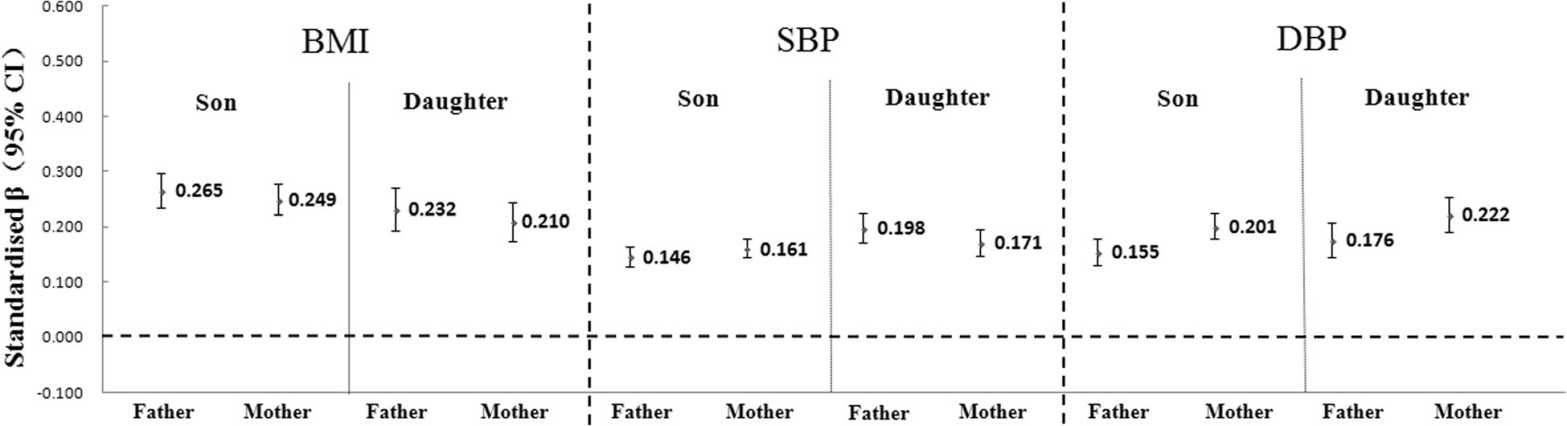
**Adjusted parent-offspring correlations (standardized coefficients and their 95% confident intervals) of BMI, SBP and DBP #.** # Each model was adjusted for children’s age, both children’s and parents’ education level and occupation.

The strength of association between maternal and paternal BMI (or BP) and offspring BMI (or BP) was also compared. As displayed in Figure 1, although adjusted parent-offspring correlations and their 95% confident interval overlapped, father-offspring correlation of BMI was slightly stronger than that of mother-offspring; For DBP, possible effect of mothers was slightly stronger than that of fathers, conversely.

## Discussion

From a large and national representative sample of Chinese nucleus families, our study presented variation of BMI and BP levels among two generations across the whole country; and demonstrated the statistically significant family aggregation of overweight/obesity in rural areas, while high BP was present in low income families and regions of all modernization levels. Furthermore, adjusted BMI level of sons and daughters showed an increasing trend across families with more overweight/obese parents, and similar trend was observed for SBP and DBP. However, same-sex parents-offspring correlations of BMI, SBP and DBP were not observed stronger than those for mother-son and father-daughter.

### Influence of economic and modernization level on BMI and BP of family members

Obesity and hypertension are important threat to health. To our knowledge, obesity and hypertension are closely related to lifestyles, which are mostly influenced by economic and modernization level of living environment [14]. A US study has reported by Schmeiser et al. that the increase in family income could explain 10%-21% of the increased BMI and 23%-29% of the increased obesity prevalence among low-income women and men [19], which indicated a positive correlation between BMI and family economic level in population with low socio-economic status in the US. Meanwhile, our study showed increasing trends of BMI in fathers, mothers and sons, and an increasing trend of SBP in fathers with family income increased in a Chinese population aged 18–64 years. However, situations might be different for other populations. For instance, Murasko et al. found a negative relationship between income and BMI among US schoolchildren aged 6–14 years [20], and Lee et al. observed the elders with high income had better control of their BP than those with low income [21]. Moreover, interestingly, we found declining trends of BMI and BP in daughters, reversely to trends of other family members. These converse correlations might attribute to less living pressure and more positive attitude to life, which come along with better economic condition. For example, the elders with higher income were engaged in less household activity and more leisure time for physical activity [21], and young women living in better economic environment were more concerned about keeping fit [22].

On the other hand, it has been discussed that increasing in prevalence of NCD risk factors are at first apparent within the most educated and wealthiest sectors of society [23]. In this study, we took regional modernization level into consideration, and observed higher BMI and BP levels in cities, especially in major ones, rather than in rural area. And these findings could be helpful in understanding the synchronization of increased prevalence of NCD risk factors and regional development and modernization, which are frequently accomplished by detrimental changes in diet and lifestyle.

### Familial aggregation of BMI, SBP and DBP

It has been already confirmed by twin studies that both genetic and shared environment factors contribute to etiology of chronic disease risk factors [24,25]. It is known that familial aggregation could comprehensively indicate known or unknown genetic and shared environmental impacts on BMI and BP of family members [13,26-30], and such familial influence could be detected from early childhood onward [29,31]. From other family-based researches, the Framingham Heart Offspring Cohort study reported ICCs of 24% and 17% for BMI and SBP among adult siblings [26], and similar results were shown as well in a Chinese siblings study conducted in Anhui province [27]. Although incomparable to those reported in other literature, we provided family relationship of father, mother and child other than siblings, and presented ICCs of BMI, SBP and DBP as 3.8%-7.4%, 4.7%-8.8% and 6.2%-11.7% across families with all income levels and regions of all modernization levels, respectively.

However, uncertainty still remains as how much of these NCD risk factors could be attributed to genetic susceptibility and how much to environmental risk [32]. Previous studies have shown the greatest absolute BMI increase was within the already heaviest population [33], and only a moderate amount of SBP and DBP is accounted for by genetic factors [34], indicating the unignorable large impact of shared environment. Recently, Brown et al. further added that, based on a US sibling cohort, sharing the same household could facilitate the social network influence on correlations in BMI [35]. Meanwhile, in our study, despite relatively low BMI, SBP and DBP levels in rural areas and low income families, statistically significant ICCs of overweight/obesity (BMI ≥ 25 kg/m^2^) were only shown in rural areas, and those of high BP (SBP ≥ 120 mmHg or DBP ≥ 80 mmHg) were presented in low-income families and in regions of all modernization levels. From a new and broader perspective, these variations of familial resemblance across different family and different regions implied both social and familial environmental influence on familial resemblance in Chinese population, probably exceeding the impact of genetic.

### Parent-offspring correlations of BMI, SBP and DBP

Parent-offspring correlations of BMI and BP are regarded to be important in recent years, which implies the possible effect of genetic and shared environment. From a longitudinal study of 226 nucleus UK families, Perez-Pastor et al. not only found a marked influence of maternal and paternal BMI on the children’s weight gain, but also showed assortative weight gain in mother-daughter and father-son pairs [10]. This same-sex association of BMI might imply shared environment rather than shared genes because selective mother-daughter and father-son gene transmission is not a common Mendelian trait. To test this hypothesis, other researchers repeated Perez-Pastor et al.’s analyses in a much larger UK cohort based on 4,654 complete parent-offspring trios, but did not add any compelling reason to integrate the belief that there were large differences in parent-offspring BMI associations with obesity prevention strategies [11]. However, despite the inconsistence findings, stronger associations of BMI and BP were frequently reported between mother and offspring [12,28,36,37], especially between mother and daughter [12,37], therefore mother is widely believed to play an important role in parent-offspring correlations, which might due to gestation [38] and their greater impact on children’s dietary behavior [37]. Although this maternal impact has also been shown in small populations of 134 Korean families [37], our study, with much larger sample size, did not find out substantial differences between father-offspring and mother-offspring correlations in Chinese. Hence, there is insufficient evidence to draw conclusion about parent-offspring associations in Asia population, and further researches are needed.

In addition, consistent with results from a Finland study [36], it was observed in our participants that BMI levels of adult children living with both overweight/obese parents were higher than those living with only one overweight/obese parent, and much higher than those from families with both normal-weight parents; similar increasing trends of sons’ and daughters’ SBP and DBP levels were shown with number of high-blood-pressure parents increased. Furthermore, among families with only one overweight/obese parent or only one high-blood-pressure parent, children’s BMI, SBP and DBP levels did not differ much according to their parents’ gender. And these findings, in our opinion, might call attention to both key roles of mother and father in primary prevention of obesity and hypertension.

### Limitations

There are several limitations in this study. Firstly, as a cross-sectional study, it was limited for us to make casual inferences; however, change of body weight and BP from normal to abnormal usually happens through a long-term process, hence the BMI and BP levels at the time of our investigation could represent a long time status of our participants, which indicates parent-offspring correlations from our study could represent a long-term interaction between two generations. Also, this study was conducted ten years ago and could not reflect the current situation in China; but this is the latest nation-wide investigation based on a large sample of Chinese Han families, which enabled us to explore not only familial aggregation and parent-offspring correlations of BMI and BP, but also its variation across families with different income levels or across regions of different modernization levels. Additionally, due to lack of information on lifestyles and on hypertension medication, it was unable to demonstrate the impact of parents on children’s health related behaviors in our population, and also unable to estimate the treatment effect on BP measurement and classification, thus future studies should focus on this issue to help better understanding the parent-offspring correlations with respect to common NCD risk factors.

## Conclusion

Family aggregation of overweight/obesity and high BP existed in rural areas and low-income families in China, respectively, suggesting both social and familial environments, alongside the impact of genetic, are important factors for NCD risk factors. Furthermore, within two generations, considering offspring with highest BMI and BP were found to live with parents both having higher-than-normal BMI and BP, and strong father-offspring and mother-offspring correlations of BMI were existed without substantial differences, both mother and father are indicated to play important roles in primary prevention strategies, and there might be great potential of family-based intervention against obesity.

## Abbreviations

BMI: Body mass index; BP: Blood pressure; SBP: Systolic blood pressure; DBP: Diastolic blood pressure; NCD: Non-communicable disease; ICC: Intra-class correlation.

## Competing interests

The authors declare that they have no competing interests.

## Authors’ contributions

YH, LH and YHH conceived of the study, completed all statistical analyses, and drafted the manuscript; YFW and GSM participated in formulating the study, collecting and interpreting the data, and helped to draft the manuscript. All authors have read and approved the final manuscript.

## Pre-publication history

The pre-publication history for this paper can be accessed here:

http://www.biomedcentral.com/1471-2458/13/686/prepub
